# Supporting social emotional learning and wellbeing of displaced adolescents from the middle east: a pilot evaluation of the ‘forward with peers’ intervention

**DOI:** 10.1186/s12888-024-05544-2

**Published:** 2024-03-04

**Authors:** Ilana Seff, Lindsay Stark, Ali Ali, Danielle Sarraf, Wafa Hassan, Carine Allaf

**Affiliations:** 1 Brown School, Washington University in St. Louis, One Brookings Drive, 63130 St. Louis, MO Box 1196, USA; 2 School of Social Work, University of Michigan, Ann Arbor, MI USA; 3 Global Educational Excellence Schools, Ann Arbor, MI USA; 4 Qatar Foundation International, Washington, DC USA

**Keywords:** Adolescents, Refugees, Wellbeing, Resilience, Evaluation, Social and emotional learning

## Abstract

**Background:**

A growing literature points to the critical role schools can play in promoting improved psychosocial wellbeing and resilience among first- and second-generation Arab immigrant and refugee adolescents, but few evaluations have examined the effectiveness of culturally adapted, school-based interventions.

**Methods:**

We conducted a pilot evaluation of a culturally adapted social and emotional learning and life skills program, Forward with Peers (FwP), and examined its potential effectiveness for this population. FwP was evaluated across three high schools in the Detroit Metropolitan Area. Within each school, one Arabic class was randomly assigned to receive FwP programming and another served as a control. The pilot evaluation sought to examine changes in several mental health and psychosocial outcomes of interest.

**Findings:**

Improvements in overall perceived social support (*P* = 0.045) and perceived social support from someone special in one’s life (0.042) were statistically significant in the treatment as compared to the control group. Comparative improvements were also marginally significant for resilience (*P* = 0.095) and perceived social support from family (*P* = 0.074).

**Conclusions:**

Findings highlight the potential of FwP and support the growing interest in establishing efficacy of school-based, culturally appropriate SEL programming to improve psychosocial wellbeing among Arab refugee and immigrant adolescents. FwP’s demonstrated improvements in resilience and social support have the potential to prevent mental health disorders and bolster coping mechanisms to minimize adverse consequences in this vulnerable population. Employing a strengths-based approach, FwP offers an alternative intervention to traditional treatment-oriented supports for the proliferation of mental health disorders within this vulnerable population.

## Background

After fleeing political conflict and violence at home, forced migrants often endure compounding stressors spanning the migration process, ranging from a lack of basic resources and services to detention to an elevated risk of further violence and abuse [1]. Refugees, asylum-seekers, and other immigrants who resettle in the United States may be forced to not only process their loss of home and experiences of migration, but also adjust to a new language and culture and learn to navigate complicated legal and public service systems [2]. The rapidly growing population of Arab-Americans, specifically, face unique migratory challenges in the U.S., including a context of hostility marked by Islamophobia and anti-immigrant sentiment. This discrimination, stigmatization, and marginalization presents significant acculturative stressors, which necessitate tailored research and intervention approaches [3, 4].

Arab refugee and immigrant adolescents, including those recently resettled to the U.S., face additional and intersecting stressors due to their age and developmental stage. Adolescence is an especially critical stage in the life course, marked by substantial change, shifting social roles and expectations, and pronounced biological and cognitive development [5]. As such, physical, neural, and psychological growth are readily influenced by external factors during these formative years, necessitating early intervention in order to minimize the negative impact of these experiences on long-term health and wellbeing [5]. Specifically during this period, Arab refugee and immigrant adolescents not only encounter repeated exposure to violence, trauma, and other adversities, but also lost years of schooling, routine discrimination, micro-aggressions, bullying, and loss of social supports [6]. They must also adapt to new social networks, languages, cultural environments, and school systems [2, 7, 8].

Combined migration stressors thus place these students at increased risk for poor mental health and compromised educational outcomes in the U.S in comparison totheir peers, affecting their overall wellbeing [6, 9]. Scholars have documented comparatively higher levels of post-traumatic stress, anxiety, depression, emotional and behavioral issues, sleep disorders, social withdrawal, and inattention among Arab refugee and immigrant adolescents [2, 4, 6–8]. A recent study examining predictors of suicide ideation and resilience notably found that being born outside of the U.S., and specifically from the Middle East and North Africa (MENA) region, was associated with increased risk of suicide ideation [9]. Responding to adolescent stress is of utmost importance, as life stressors and perceived stress are the most widely identified risks factors for suicidality in this population [10].

Practitioners and researchers alike have become increasingly interested in the role of schools in promoting healthy development, improved psychosocial wellbeing, and enhanced resilience among Arab refugee and immigrant adolescents [11, 12]. Given that adolescents spend most of their time in schools and there exists built-in infrastructure for support and early identification of mental illness, schools may offer an accessible, familiar, and non-stigmatizing setting for targeted adolescent refugee and immigrant psychosocial interventions [12, 13]. School-based programming has been found to have enormous potential for preventing the onset of adverse mental health outcomes and contains the ability to promote the critical protective factors of school belonging and resilience [14].

Certain school- and organization-based interventions have been gaining popularity in supporting refugee and immigrant populations and bolstering protective factors. Schools in high income countries have been investing in social and emotional learning (SEL) programs in recent years as an effort to strengthen supports for resettled populations [15]. SEL programs employ approaches within the non-cognitive domain and teach skills outside of the academic sphere, striving to develop adolescent self-awareness, self-management, relationship skills, and responsible decision making [15, 16]. A limited number of studies have found that SEL programming has sustained a more supportive environment for adolescent refugees and immigrants–especially those arriving from conflict-affected contexts–who benefit from increased attention to self-awareness construction and development of supportive, caring relationships among students and staff [17, 18].

Yet, on a broad scale, mental health interventions for refugee and immigrant adolescents remain sparse and those that do exist remain largely understudied, unmeasured, and unreported. Additional research on best practices for culturally adaptive program implementation and measurement of outcomes are necessary to inform the development of guidance for best practice [12, 13]. This study explores the feasibility and potential effectiveness of an integrated SEL and life skills program, Forward with Peers (FwP). The life skills components of the program include content that students may not otherwise be exposed to, including guidance on leadership and bullying, as well as introductions to financial, computer, and career skills. We conducted a quasi-experimental pilot evaluation of the program with first- and second-generation immigrant and refugee students from the MENA region to understand program effect on student resiliency, social support, hope, and school belonging.

## Methods

### Study setting

Data were collected as part of the Study of Adolescent Lives after Migration to America (SALaMA), a multi-city, mixed methods study assessing the mental health and psychosocial needs of Arab refugee and immigrant adolescents [19]. The FwP program was implemented in the Detroit Metropolitan Area (DMA), Michigan. Over many years, the DMA has become a highly preferred settlement area for MENA immigrants, making it the largest Arab and Arab-American ethnic and cultural enclave in the United States [20]. Nearly 50% of the population in Dearborn, a city in the DMA, identifies as Arab [21]. As such, DMA schools contain a high percentage of immigrant, refugee, and asylum-seeking students.

### Forward with peers

Forward with Peers (FwP) is a 10-week school-based program that aims to create a safe place to further culturally adaptive social and emotional learning and build students’ toolset to achieve their academic, professional, and personal goals. The program was designed to address important SEL and life skills topics, as well as reduce stigma around mental health needs and help-seeking in this refugee and immigrant population. Content was drawn from the Collaborative for Academic, Social, and Emotional Learning (CASEL), which aims to strengthen pathways between resilience, hope, and a sense of belonging within peer groups and schools [22]. Tailored lessons and activities support students in building a stronger sense of identity, developing new friendships, gaining leadership skills, planning for college and careers, learning about available resources, and practicing self-care and coping strategies. Session topics include mental health, cultural awareness, leadership, bullying and relationships, financial literacy, computer skills, and career exploration, each tailored to promote evidence-based protective factors for adolescent immigrants (Table 1). Several key program features were also included to maximize engagement for this target population, including incorporating gender sensitivity to align with participants’ perceived norms of interaction, adopting trauma-informed approaches to guide discussions and activities, demonstrating cultural sensitivity by acknowledging holidays, prayer times, dietary restrictions, and clothing preferences, and acknowledging and respecting religious and ethical considerations voiced by the participants.

**Table 1:** An overview of the program sessions

Week	Session Topic	Session Description
1	Welcome Session	Introduction to the FwP program, review of the syllabus, establishment of group norms and expectations, and ice breaker activities to help participants get to know each other.
2	Mental Health	Facilitated discussions around mental health, well-being, and stress management techniques to help participants develop an understanding of their emotions and how they may impact their behavior. Aims to reduce mental health stigma and overcome fear of seeking care.
3	Cultural Awareness	Discussions on critical topics such as gender equality, discrimination and racism, stereotypes, and norms. An interactive presentation is given, and participants are encouraged to share their opinions and experiences.
4	Bullying & Relationship Building	Discussions revolve around various types of bullying, building supportive relationships, and using healthy coping mechanisms. Participants are encouraged to share personal experiences and develop strategies to stop bullying. Session conclude with participants creating initiatives to be carried out by the group.
5	Leadership	Activities teach participants team building and group process, effective communication, goal setting, problem solving, and decision making. The attributes and skills of a good leader are identified by analyzing examples and then applying them in a real-life scenario.
6	Computer Basics	Introduction to Microsoft Office Suite and Google Suite, online safety, and a facilitated discussion on how to use these tools to enhance education and career development. Using a combination of the tools shared, participants create a multimedia project, such as a video, poster, or presentation.
7	Financial Literacy	Finance-related discussion and activity topics include: loans, credit and debit cards, credit scores, investments, options to fund college tuition, and budgeting.
8	“Real Life” Project	After selecting a profession, participants create and present a monthly budget based on their profession’s income.
9	Career Exploration	Lesson on career planning, searching for colleges and jobs, the college application process, completing applications, and interview strategies and techniques. Participants create resumes with guidance and feedback from the facilitator, which can be used by participants in their future endeavors.
10	Goals	Participants create individual goal posters, which they share in both small and large groups. The session ends with reflections and take-aways from the program.

**Table 2:** Descriptive statistics at baseline

	Full sample (***n*** = 108)	Control (***n*** = 39)	Treatment (***n*** = 69)	***P***-value
Age	14.98 [1.01]	15.03 [1.06]	14.96 [0.98]	0.733
Gender				0.896
Girl	43 (40.19%)	16 (41.03%)	27 (39.71%)	
Boy	60 (56.07%)	22 (56.41%)	38 (55.88%)	
Other	4 (3.74%)	1 (2.56%)	3 (4.41%)	
Born in MENA region	46 (41.44%)	15 (38.46%)	30 (44.12%)	0.527
Paid job				0.146
Yes	17 (15.89%)	10 (25.64%)	7 (10.29%)	
No	81 (75.70%)	26 (66.67%)	55 (80.88%)	
Prefer not to say	9 (8.41%)	3 (7.69%)	6 (8.82%)	
Hope (CHS)	4.37 [1.01]	4.47 [0.89]	4.32 [1.09]	0.476
Resilience (CYRM-12)	31.02 [4.11]	31.97 [3.08]	30.52 [4.52]	0.127
Loneliness	40 (37.74%)	21 (55.26%)	19 (27.94%)	0.012
Suicide ideation	10 (95.2%)	4 (10.53%)	6 (89.6%)	0.853
Perceived school belonging (PSSM)	3.77 [0.79]	3.72 [0.85]	3.81 [0.76]	0.564
Perceived Social Support (MSPSS)	5.21 [1.63]	5.40 [1.42]	5.09 [1.74]	0.343
Significant other (special person in one’s life)	5.25 [1.77]	5.44 [1.53]	5.13 [1.90]	0.390
Friends	5.13 [1.67]	5.35 [1.42]	5.00 [1.81]	0.309
Family	5.24 [1.82]	5.43 [1.57]	5.13 [1.96]	0.424

Values are mean [sd] or n (%). *P*-Values are presented from chi-squared tests to examine difference between groups at baseline

**Table 3:** Difference-in-difference analysis

	Change for control group	Change for treatment group	DiD	***P***-Value
Hope (CHS)	0.000	-0.060	-0.061	0.761
Resilience (CYRM-12)	-0.136	0.145	0.281^┼^	0.095
Loneliness	-0.038	0.002	0.039	0.815
Suicide ideation	0.113	-0.016	-0.130	0.556
Perceived school belonging (PSSM)	0.046	0.070	0.023	0.890
Perceived social support (MSPSS)	-0.205	0.184	0.390*	0.046
Significant other (special person in one’s life)	-0.215	0.195	0.410*	0.042
Friends	-0.143	0.161	0.304	0.131
Family	-0.163	0.187	0.350^┼^	0.074

Difference-in-difference estimates are statistically significant at ^┼^*P* < 0.1; *P < 0.05; **P < 0.01.

FwP sessions were structured to last approximately one hour, aligning with the duration of a standard class period, and took place once a week during the school day. The co-creator of the program, who is a trained facilitator, led each session in English. However, as a native Arabic speaker, the facilitator was able to translate content into Arabic if and as needed.

### Study participants and procedures

The pilot evaluation took place across three high schools in the DMA. Given the pilot nature of the study, sample size calculations were not conducted; within each school, two Arabic classes were selected for inclusion in the study. Classes were chosen based on scheduling availability of teachers and the program facilitator and in such a way as to maximize composition similarities between the two classes. Study classes included students at all levels of Arabic proficiency as well as students born both in the U.S. and in the MENA region. Class sizes ranged from 12 to 20 students and were mixed-gender. Within each of the three schools, one class was randomly assigned to receive FwP programming and the other was assigned to the control arm.

Quantitative survey questionnaires were administered to students in both study arms one week before the start of the intervention and one week after its completion. Parental consent for participation in the study was obtained online prior to baseline data collection. After obtaining informed assent from students on the day of baseline data collection, students were assigned a study ID that they entered into the survey form in order to link student responses between baseline and endline. Students’ names were not tied to their survey responses. Students took the survey on Qualtrics with a member of the research team present to answer any questions that arose. Students were able to take the survey in either English or Arabic, depending on their preference. Surveys took approximately 15–25 min to complete. Students received a $15 incentive for participation in each of the baseline and endline data collections.

All study procedures received ethical approval from the Institutional Review Board at Washington University in St. Louis (IRB ID # 202,108,107).

### Survey measures

The pilot evaluation sought to examine changes in several mental health and psychosocial outcomes of interest, described below.

#### Resilience

The Children and Youth Resilience Measure-12 (CYRM-12) was used to examine resilience [23]. This measure has been validated with adolescent Syrian refugees [24]. A modified version was employed, whereby respondents were prompted to answer *No (1)*, *Sometimes (2)*, *or Yes (3)*  for 12 statements, such as “I try to finish activities that I start,” and “I know where to go to get help.” The final score reflects a sum of all items and can take a value from 12 to 36 (Cronbach’s alpha = 0.81).

#### Symptoms of distress

Feelings of loneliness and suicide ideation were also measured using one question each. Respondents were asked how often they felt lonely and how often they had thoughts of ending their life in the last month. Measures of loneliness and suicide ideation were operationalized in the binary, whereby those who reported either symptom at least sometimes in the last month received a ‘1’ and those who did not received a ‘0.’

#### Social support

The Multidimensional Scale of Perceived Support (MSPSS), which has previously been validated with migrant students [25], was used to assess social support [26]. The 12-item scale also includes three sub-scales that measure perceived social support from a significant other (referenced as a “special person” in the survey), friends, and family. Respondents provide their level of agreement with each statement using a 7-point Likert scale ranging from *Very strongly disagree (1)*  to *Very strongly agree (7)*. An average across all twelve items is calculated for the final score, which can take a value of 1 to 7, whereby higher scores reflect greater levels of social support. Cronbach’s alpha for the full scale was 0.97.

#### Hope

Participants’ feelings of hope were measured using the Children’s Hope Scale (CHS) [27], which has been previously validated with adolescent refugees [28]. Respondents are presented with six statements, such as “I can think of many ways to get the things in life that are most important to me,” and “I think I am doing pretty well,” and are asked to respond with a 6-point Likert scale that ranges from *None of the time (1)*  to *All of the time (6)*. The final score reflects the average score across all six items and may assume a value from 1 to 6, where 6 indicates greater hopefulness. The CHS exhibited good internal consistency, with a Cronbach’ alpha of 0.82 at baseline.

#### School belonging

Perceived school belonging was measured using the Psychological Sense of School Membership (PSSM) [29]. Respondents are presented with 18 statements, such as “I feel like a real part of my school,” and “People at this school are friendly to me,” and asked to indicate their level of agreement using a 5-point Likert scale that ranges from *Completely disagree (1)*  to *Completely agree (5)*. Following an approach employed in other studies, negatively-phrased items were removed to improve internal consistency. The final scale is calculated as the mean of all item responses and can have a value from 1 to 5, where higher values reflect greater perceived school belonging. Cronbach’s alpha for the positively phrased items within the PSSM was 0.89.

The survey questionnaire also collected information on participants’ age, gender, country of birth, and whether they held a paid job.

### Analysis

Summary statistics for sociodemographic variables and outcomes of interest were calculated for the full sample and within both the treatment and control groups at baseline. Balance in all variables at baseline between the two groups was assessed using t-tests. To estimate whether FwP had a statistically significant effect on outcomes of interest for the treatment group as compared to the control group, a difference-in-difference approach was employed [30]. A difference-in-difference approach controls for time-unvarying factors between baseline and endline, but not those that may vary over time. As such, our difference-in-difference approach assumes that all students in the study would have exhibited similar trends in the outcomes of interest in the absence of any intervention.

## Results

A total of 69 students participated from the intervention arm and 39 students participated from the control arm, out of approximately 112 students. Table 2 presents summary statistics for the full sample as well as within the control and treatment arms. Study participants were an average of 14.98 years old and 40.19% identified as girls, 56.07% as boys, and 3.74% as ‘other.’ Slightly over 40% of the full sample was born in the MENA region and 15.89% had a job for which they were paid at baseline. Excluding feelings of loneliness, all other outcomes of interest were balanced at baseline. Participants in the control group exhibited greater self-reported prosocial behaviors (*P* < 0.001) and feelings of loneliness (*P* = 0.012) as compared to those in the treatment group.

**Fig. 1:**
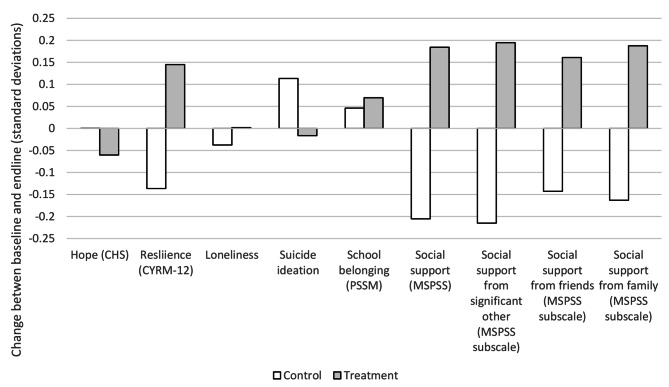
Changes in outcomes of interest between baseline and endline, standardized

Figure 1 demonstrates the changes in outcomes of interest between baseline and endline for both control and treatment groups. Outcomes of interest are standardized for ease of presentation. All outcomes of interest, excluding hope, improved or remained effectively unchanged for the treatment group and worsened or remained effectively unchanged for the control group. The difference-in-difference analysis also revealed statistically significant comparative improvements in several outcomes of interest for the treatment as compared to the control group (see Table 3). Specifically, the improvements in overall perceived social support (*P* = 0.045) and perceived social support from someone special in the student’s life (0.042) were statistically significantly larger in the treatment as compared to the control group. The comparative improvements between the treatment and control groups were also marginally statistically significant for resilience (*P* = 0.095) and perceived social support from family (*P* = 0.074).

## Discussion

This pilot evaluation of FwP revealed promising findings for the program’s capacity to improve first- and second-generation Arab refugee and immigrant mental and psychosocial well-being. Specifically, participants in the treatment arm saw statistically larger increases in overall perceived social support and perceived social support from someone special in their life, as compared to those in the control arm. Changes in resilience and perceived social support from family were also marginally statistically significant between the two groups. Further, we discovered positive trends for other outcomes of interest, including decreases in loneliness and suicide ideation and improvements in school belonging.

Historically, traditional programming for adolescent refugees and immigrants has focused on their exposure to trauma, emphasizing the need to “fix” the consequent impacts on mental health [31, 32]. While these treatment-oriented supports can be beneficial for adolescent refugees and immigrants, these interventions often fail to recognize the resilience, strengths, and lived experiences of their target recipients. As such, researchers and practitioners supporting adolescent refugees and immigrants have increasingly advocated for a strengths-based approach that promotes positive contextual, social, and individual factors and builds on existing strengths for healthy adolescent development [33]. Research has shown that, through targeting protective and promotive factors in adolescents’ social ecologies, strengths-based approaches may bolster agency, resilience, and mental wellbeing [34].

Drawing on a strengths-based approach, FwP supports participants’ capacity to harness their resilience, future-oriented planning, leadership skills, and coping strategies. For example, the program’s “Real Life” Project gives students full creative range to create and budget a future career and lifestyle for one month, encouraging them to think about their future goals, strengthen their problem-solving skills, and practice decision-making. Similarly, the Leadership session cultivates students’ skills around team building, group process, and effective communication. These sessions, along with the others included in FwP, aim to remind students of and strengthen the individual assets and resources they have access to. As suggested by our data and existing literature, cultivation of these skills may both strengthen resilience and perceived social support in the face of adversity, ultimately leading to improved overall psychosocial wellbeing [9–11].

Observed improvements in perceived social support may also be a result of FwP’s incorporation of transformative SEL principles. Since 2019, school practitioners have increasingly advocated for a shift from the conventional SEL curriculum to “transformative SEL,” a “form of SEL implementation where young people and adults build strong, respectful, and lasting relationships to engage in co-learning” and engage in “critical examination of individual and contextual factors that contribute to inequities and collaborative solutions that lead to personal, community, and societal well-being” [35]. To holistically foster wellbeing, transformative SEL programs focus on recognizing adolescents’ voices and participation, acknowledging the importance of cultural resources, and, ultimately, building their feelings of belonging [35, 36]. Such programs incorporate the development of positive ethnic-racial identity and facilitate critical analyses of privilege and power within SEL competencies [27–37]. Relatedly, increased socialization more broadly, including the promotion of friendships and social support as resources, may lead to greater senses of belonging for this population. Improvements in perceived school belonging increased across both the treatment and control groups over the course of the intervention. Given that the program was implemented as students were beginning to return to school following COVID school closures, this finding may reflect the psychosocial benefits of in-person versus online schooling, as demonstrated in other post-COVID studies [38, 39].

For Arab adolescent refugees and immigrants in the US, stigma and perceived threats to reputation may discourage students from seeking formal mental health services or discussing mental health with their parents [40]. Further, inattention to matters of identity, belonging, and inequality in schools has been shown to result in a variety of negative consequences, including increased acculturative stress and a compromised development of a positive ethnic-racial identity [3]. Evidence has shown the deleterious effects of an “assimilationist” stance in schools on adolescent refugee and immigrants’ sense of school belonging and academic success, undermining their sense of self-efficacy and ability to manage acculturative stressors [36, 41]. Alternatively, refugee and asylum-seeking adolescents from the MENA region have responded positively when their schools have recognized their cultural identities as assets and refrained from reducing them to stereotypes of marginalized and traumatized people [2, 41]. Research suggests that this positive sense of ethnic-racial identity can help students to develop and employ “cultural repertoires” that allow them to build and sustain self-esteem and a strong sense of belonging, ultimately leading to resilience against marginalization [4]. Thus, transformative SEL programs work to acknowledge these significant cultural contexts to enhance self-efficacy and belonging.

FwP builds on these principles to foster students’ identity dimensions as they relate to ethnicity, religion, country-of-origin, and others, with an emphasis on how cultural norms associated with these identities may influence student mental health and wellbeing [36]. Stemming from participant, teacher, and staff feedback, FwP sessions are facilitated with a trauma-informed approach that acknowledges students’ diverse cultural and religious practices and encourages student-guided discussions and projects. A trauma-informed approach posits the assumption that an individual is more likely than not to have a history of trauma; thus, services are organized appropriately to understand how this trauma impacts their daily existence, offer necessary support, and take steps to avoid re-traumatization [42]. As such, our findings and the literature point to the potential of transformative SEL programs to expand social and emotional learning and further incorporate attention and resources to issues of acculturation, cultural responsiveness, and peer relationships that foster inclusion and belonging [36].

Findings from this pilot evaluation should be considered alongside a few limitations. First, due to resource constraints associated with conducting a small pilot evaluation, as well as partner schools’ capacity, our evaluation included only three classes in each study arm. Future evaluations should build on these preliminary findings, ensuring fully powered sample sizes both at the cluster (classroom) and participant (student) levels. While this pilot evaluation shows promise for FwP’s ability to improve wellbeing outcomes for Arab immigrant adolescents, both a larger sample and a longer follow-up period would allow for a greater understanding of sustained impacts and potential underlying mechanistic pathways. Finally, while this program suggests promise for Arab-American students, it might be usefully expanded to consider its potential for a broader immigrant population.

## Conclusion

While ample evidence exists on the efficacy of school-based SEL programs and strengths-based approaches, few programs have been evaluated and measured to understand their effects on adolescent wellbeing and form guidance for best practice for programming. Given the adverse effects of migration, there exists a critical need for targeted interventions to promote mental health and psychosocial wellbeing, bolster sources of resilience, and encourage help-seeking among first- and second-generation Arab refugee and immigrant students. The findings from this pilot evaluation of FwP, a social and emotional learning and life skills intervention, document the program’s potential to bolster social supports and resilience. Given the promising findings from this pilot evaluation of FwP, more robust measurement is needed to further understand FwP and other similar interventions’ ability to promote protective factors for Arab refugee and immigrant adolescents. This program may also act as a model for future implementations to promote resilience, social support, and school belonging among adolescent refugees and immigrants from the MENA region.

## Data Availability

The datasets supporting the conclusions of this article can be found at https://sites.wustl.edu/salama/data/.
